# Choosing the right instruments for psychotrauma related research

**DOI:** 10.3402/ejpt.v6.30585

**Published:** 2015-12-28

**Authors:** Miranda Olff

Choosing the right instrument for trauma research or the best clinical measure to assess a patient's mental health status is not an easy task. What would be the best measure to assess trauma history, posttraumatic stress symptoms, depression or anxiety symptoms, addiction problems, or other frequent consequences of trauma? And which are the risk or resilience factors that we need to address and how? Should it be a clinical interview or would a self-report measure be more appropriate? Is paper-and-pencil to be preferred or an online tool or mobile app even (Olff, 2015)?

**Table 1:** Suggested “golden standard” mental health instruments for trauma-related assessments that are freely available, valid, and relatively short^a^

Topic	Scale	To obtain scale	©/Reference
Potential Traumatic Events Checklist (17 items)	Life Events Checklist for DSM-5 (LEC-5)	http://www.ptsd.va.gov/professional/assessment/te-measures/life_events_checklist.asp	**©**Weathers, Litz, Keane, Palmieri, Marx, & Schnurr—National Center for PTSD (2013)
Posttraumatic Stress Quick screener (5 items)	The Primary Care PTSD Screen for DSM-5 (PC-PTSD 5)	http://www.ptsd.va.gov/professional/assessment/screens/pc-ptsd.asp and http://www.ptsd.va.gov/professional/assessment/DSM_5_Validated_Measures.asp	**©**Prins et al.—National Center for PTSD (2013)
Posttraumatic Stress Questionnaire PTSD symptoms (20 items)	PTSD Checklist for the DSM-5 (PCL-5)	http://www.ptsd.va.gov/professional/assessment/adult-sr/ptsd-checklist.asp	**©**Weathers, Litz, Keane, Palmieri, Marx, & Schnurr—National Center for PTSD (2013)
Posttraumatic Stress Interview	Clinician-Administered PTSD Scale for DSM-5 (CAPS-5)	http://www.ptsd.va.gov/professional/assessment/adult-int/caps.asp	**©**Weathers, Blake, Schnurr, Kaloupek, Marx, & Keane—National Center for PTSD (2013)
Psychological Resilience Brief questionnaire (10 items)	Resilience Evaluation Scale (RES)	E-mail: Christianne van der Meer, c.a.meervander@amc.uva.nl Hans te Brake, h.te.brake@arq.impact.org	**©**AMC & Arq (2013) Van der Meer, Te Brake, Bakker & Olff. (2015)
Depression, Anxiety, Stress Questionnaire (21 items)	Depression Anxiety Stress Scales-21 (DASS-21)	http://www2.psy.unsw.edu.au/dass/down_W6.htm	**©**Lovibond, School of Psychology, University of New South Wales, Sydney
Peritraumatic Distress Questionnaire (13 items)	Peritraumatic Distress Inventory (PDI)	http://www.info-trauma.org/flash/media-e/triageToolkit.pdf	Brunet, et al. (2001).
Peritraumatic Dissociation Questionnaire (10 items)	Peritraumatic Dissociative Experiences Questionnaire (PDEQ)	http://www.info-trauma.org/flash/media-e/triageToolkit.pdf	Marmar, et al. (1997).

a We aim to extend this list, and we would be happy to receive input.

To be able to compare data collected across labs and countries, combining data sets for meta- or mega-analysis using standardized tools would be a major accomplishment. In Table 1, in order to move toward this goal, a selected set of instruments is listed per domain; they are valid and reliable and freely available, as well as relatively quick to administer. This list is based on our work for a European Union (EU)-funded project (www.OPSIC.eu), where a large set of instruments was evaluated together with other consortium partners. In another EU-funded project, INPREZE, we have developed a mobile app for assessing trauma-related symptoms (Smart Assessment on your Mobile (SAM); see Olff, 2015), which is also based on this type of valid, relatively brief instruments. We aim to extend this list with other instruments and to add the language it is available in. We will be happy to receive your input.

In this journal, we have published on a wide range of measures on adults (see Table 2) and on children and family (see Table 3) that may help individuals make more informed choices. *European Journal of Psychotraumatology* has now created a section on *Instruments* where all articles will be placed that address psychometric tools or trauma-related measures (such as those in Table 2 and 3) regardless of the primary category of papers they fall into, for example, clinical practice articles and basic research articles.

**Table 2:** EJPT articles on psychometric tools and instruments: Adults

*Trauma exposure*	•	How to quantify exposure to traumatic stress? Reliability and predictive validity of measures for cumulative trauma exposure in a post-conflict population. Wilker et al., 2015
*Screening*	•	Implementing a screening programme for posttraumatic stress disorder following violent crime. Bisson et al., 2010
*PTSD symptoms*	•	Validation of a French adaptation of the Harvard Trauma Questionnaire among torture survivors from sub-Saharan African countries. De Fouchier et al., 2015
	•	The validation of the Polish version of the Posttraumatic Diagnostic Scale and its factor structure. Dragan et al., 2012
	•	Cross-cultural and factorial validity of PTSD check list—military version (PCL-M) in Sinhalese language. Semage et al., 2013
	•	Validation of the Davidson Trauma Scale in its original and a new shorter version in people exposed to the F-27 earthquake in Chile. Leiva-Bianchi et al., 2013
*Secondary traumatization*	•	Psychometric properties of the Questionnaire for Secondary Traumatization. Weitkamp et al., 2014
*Dissociation symptoms*	•	The Shutdown Dissociation Scale (Shut-D). Schalinski et al., 2015
	•	Latent profile analysis and principal axis factoring of the DSM-5 dissociative subtype. Frewen et al., 2015
	•	Measuring fragmentation in dissociative identity disorder: the integration measure and relationship to switching and time in therapy. Barlow & Chu, 2014
*Anhedonia*	•	Assessment of anhedonia in psychological trauma: development of the Hedonic Deficit and Interference Scale. Frewen et al., 2012a
	•	Assessment of anhedonia in psychological trauma: psychometric and neuroimaging perspectives. Frewen et al., 2012b
*Sleep*	•	Validation of the French version of the Pittsburgh Sleep Quality Index Addendum for posttraumatic stress disorder. Ait-Aoudia et al., 2013
*Other trauma-related measures*	•	The Appetitive Aggression Scale—development of an instrument for the assessment of human's attraction to violence. Weierstall & Elbert, 2011
	•	Construction of a questionnaire for readiness to reconcile in victims of human rights violations. Stammel et al., 2013
*Mobile or online tools*	•	Mobile mental health: a challenging research agenda. Olff, 2015

The ultimate aim of collecting these “Instruments articles” is to create an authoritative multiple language resource that offers the possibility of finding the right type of measure for the right type of topic in the right language. Ideally, we would like to have free access to all instruments described, without cost or complex copyright issues.

There is ongoing discussion on whether one should stick to diagnoses as defined by classification systems, such as the American Psychiatric Association (DSM-5) or the World Health Organization's International Classification of Diseases (ICD-10), currently under revision but with release date for ICD-11 in 2018. Table 4 shows articles that address whether DSM or ICD might be the best approach toward diagnosing posttraumatic stress disorder.

**Table 3:** EJPT articles on psychometric tools and instruments adults: Child and family

Trauma exposure	•	What makes a life event traumatic for a child? The predictive values of DSM—criteria A1 and A2. Verlinden et al., 2013
Screening	•	Enhanced screening for posttraumatic stress disorder and comorbid diagnoses in children and adolescents. Verlinden et al., 2015
	•	Evaluating predictive screening for children's post-injury mental health: New data and a replication. Kassam-Adams et al., 2015
PTSD symptoms	•	A cross-cultural validation of the Clinician Administered PTSD Scale for Children and Adolescents in a Dutch population. Diehle et al., 2013
	•	The psychometric properties of the Trauma Symptom Checklist for Young Children in a sample of Swedish children. Nilsson et al., 2012
	•	Reliability, factor structure, and validity of the German version of the Trauma Symptom Checklist for Children in a sample of adolescents. Matulis et al., 2015
Adherence	•	Therapeutic adherence and competence scales for Developmentally Adapted Cognitive Processing Therapy for adolescents with PTSD. Gutermann et al., 2015
Cognitions	•	The Dutch version of the Child Posttraumatic Cognitions Inventory: validation in a clinical sample and a school sample. Diehle et al., 2015
Parents	•	Factor structure of the Parent Emotional Reaction Questionnaire: analysis and validation. Holt et al., 2015
Family	•	Development of a Childhood Attachment and Relational Trauma Screen (CARTS): a relational-socioecological framework for surveying attachment security and childhood trauma history. Frewen et al., 2013
	•	Assessing the Family Dynamics of Childhood Maltreatment History with the Childhood Attachment and Relational Trauma Screen (CARTS). Frewen et al., 2015

**Table 4:** Diagnosis of PTSD DSM-5/ICD-11/symptom structure

•	Less is more? Assessing the validity of the ICD-11 model of PTSD across multiple trauma samples. Hansen et al., 2015
•	Posttraumatic stress disorder according to DSM-5 and DSM-IV diagnostic criteria: a comparison in a sample of Congolese ex-combatants. Schaal et al., 2015
•	An evaluation of ICD-11 PTSD and complex PTSD criteria in a sample of adult survivors of childhood institutional abuse. Knefel & Lueger-Schuster, 2013
•	Evidence for proposed ICD-11 PTSD and complex PTSD: a latent profile analysis. Cloitre et al., 2013
•	Update to an evaluation of ICD-11 PTSD and complex PTSD criteria in a sample of adult survivors of childhood institutional abuse by Knefel & Lueger-Schuster (2013): a latent profile analysis. Knefel et al., 2015
•	Distinguishing PTSD, Complex PTSD, and Borderline Personality Disorder: A latent class analysis. Cloitre et al., 2014
•	Complex PTSD: research directions for nosology/assessment, treatment, and public health. Ford, 2015
•	The underlying dimensionality of PTSD in the diagnostic and statistical manual of mental disorders: where are we going? Armour, 2015
•	Symptom structure of PTSD: support for a hierarchical model separating core PTSD symptoms from dysphoria. Rademaker et al., 2012
•	Factor structure of PTSD, and relation with gender in trauma survivors from India. Charak et al., 2014

For research, it might also be of value to study phenotypes or domains that may be present across the classical disorders, such as cognitive, memory, or executive functions. NIMH has introduced Research Domain Criteria (RDoC) as a new way of studying mental disorders. It integrates many levels of information (from genomics to self-report) to better understand the basic dimensions of functioning. I welcome more research on this type of assessment in our journal.

*Miranda Olff* Editor-in-Chief
